# Phylogenetic relationships and diversity of bat-associated *Leptospira* and the histopathological evaluation of these infections in bats from Grenada, West Indies

**DOI:** 10.1371/journal.pntd.0007940

**Published:** 2020-01-21

**Authors:** Amanda I. Bevans, Daniel M. Fitzpatrick, Diana M. Stone, Brian P. Butler, Maia P. Smith, Sonia Cheetham

**Affiliations:** 1 Department of Pathobiology, School of Veterinary Medicine, St. George’s University, Grenada, West Indies; 2 Department of Public Health and Preventive Medicine, School of Medicine, St. George’s University, Grenada, West Indies; Baylor College of Medicine, UNITED STATES

## Abstract

Bats can harbor zoonotic pathogens, but their status as reservoir hosts for *Leptospira* bacteria is unclear. During 2015–2017, kidneys from 47 of 173 bats captured in Grenada, West Indies, tested PCR-positive for *Leptospira*. Sequence analysis of the *Leptospira rpoB* gene from 31 of the positive samples showed 87–91% similarity to known *Leptospira* species. Pairwise and phylogenetic analysis of sequences indicate that bats from Grenada harbor as many as eight undescribed *Leptospira* genotypes that are most similar to known pathogenic *Leptospira*, including known zoonotic serovars. Warthin-Starry staining revealed leptospiral organisms colonizing the renal tubules in 70% of the PCR-positive bats examined. Mild inflammatory lesions in liver and kidney observed in some bats were not significantly correlated with renal *Leptospira* PCR-positivity. Our findings suggest that Grenada bats are asymptomatically infected with novel and diverse *Leptospira* genotypes phylogenetically related to known pathogenic strains, supporting the hypothesis that bats may be reservoirs for zoonotic *Leptospira*.

## Author summary

Leptospirosis is a worldwide disease of humans and animals caused by pathogenic strains of *Leptospira* spp. These bacteria are transmitted in urine from infected and usually asymptomatic reservoir hosts. *Leptospira* have been detected in several species of animals in Grenada, and human cases of leptospirosis are reported annually. However, little is known about the source of infection, and while rats are the most commonly recognized reservoir host of *Leptospira*, there is growing evidence that bats may also be asymptomatic carriers in the wild. To examine this, we captured different species of bats in Grenada, West Indies, from 2015 to 2017, tested kidney tissue for *Leptospira* spp. bacteria by PCR, and performed histological examination to see whether there is any relationship between *Leptospira* infection and associated lesions. Our results suggest that two species of bats tested—*Artibeus* spp. and *Glossophaga longirostris*—are asymptomatically infected with several types of *Leptospira* bacteria that may be new to science and that are phylogenetically related to strains that are known to cause disease.

## Introduction

Leptospirosis, caused by spirochete bacteria, is the most frequently reported zoonosis worldwide with an estimated 1.03 million cases each year. Leptospirosis is a leading cause of morbidity and mortality, with tropical areas accounting for the majority of all human cases and deaths [1]. There are at least 35 recognized species and 250 serovars of *Leptospira*, and depending on the convention used, these species have been clustered into either three [2,3] or four [4] major monophyletic groups that correspond with their pathogenicity and niche. To date, at least seventeen species have been described in Group I, previously known as the “pathogenic” group. Several of these species cause severe disease and/or death in some animals and humans, while chronically infected reservoir hosts can be asymptomatic or have few symptoms of infection [5]. Humans can also become infected with some of the *Leptospira* species in Group II, also known as the “intermediately pathogenic” group, though infection is typically subclinical to mild [6,7]. Non-pathogenic, saprophytic *Leptospira* comprise either one or two clades depending on the convention used [3,4].

Pathogenic *Leptospira* colonize the kidneys of the infected host and are excreted in the urine. Infections occur through direct contact with the urine of infected animals or when this urine contaminates the environment. The bacteria enter the body through cuts or abrasions on the skin, or through the mucous membranes of the mouth, nose, and eyes [reviewed in [8]].

Bats are reservoir hosts for several important zoonotic pathogens including viruses and bacteria [9,10]. Although serological evidence of bat exposure to *Leptospira* spp. has been reported from several parts of the world [11–13], and molecular detection of infection by PCR has also been documented [14–20], the role of bats in the epidemiology of zoonotic *Leptospira* is not well-understood. *Leptospira* infection has been detected in over 50 bat species belonging to eight of the nine investigated bat families, representing bats from many geographical regions, including both the tropics and subtropics [21]. Importantly, it has also been documented that bats can carry *Leptospira* in the renal tubules and shed the spirochetes in their urine for at least five months [22]. Taken together, the global abundance of bats, their spatial association with humans and both domestic and wild animals, and evidence that bats can shed *Leptospira* in their urine suggest that bats may be epidemiologically significant for *Leptospira* transmission.

Grenada is a tropical island nation in the southern part of the West Indies. Between 2008 and 2014, Grenada reported from two to 22 cases of human leptospirosis annually [23]. This likely reflects a small fraction of actual infections, as most cases of leptospirosis in humans, regardless of geographic area, are not confirmed or reported [24,25]. Humans and animals in Grenada have tested seropositive for at least 17 serotypes of *Leptospira*. *Leptospira*-seropositive animals in Grenada include bats, cattle, chickens, goats, mongooses, pigs, sheep and toads [11,26–28]. Research performed in the 1970s in Grenada found that 13/61 (21%) *Anoura* spp. bats and 4/52 (8%) of *Glossophaga* spp. bats were *Leptospira*-seropositive. Reacting serovars included *L borgpetersenii* serovar Tarassovi; *L*. *interrogans* serovars Autumnalis, Canicola, Hebdomadis, and Icterohaemorrhagiae; *L*. *noguchii* serovars Bataviae and Panama; and *L*. *santarosai* serovar Shermani [11]. However, *Leptospira* could not be cultured from the extracted kidney tissue of any seropositive bats. Thus, in Grenada, there are no reports to date documenting active infection in bats with any *Leptospira* species whether by PCR or microscopic observation.

PCR-based tools have become essential in studying *Leptospira* biology, phylogeny, and pathogenesis, and are key to diagnosing active infection. Several genes have been used singly or in tandem to type *Leptospira* (*flaB*, *gyrB*, *rpoB*, 16SrRNA *rrs*, *secY*, *lipL32*) [29]. In particular, the *rpoB* gene is an ideal target for phylogenetic analyses: it allows for discrimination among *Leptospira* species better than most other gene sequences (e.g., *rrs*, *lipL32*), and all recognized *Leptospira* species to date have partial or whole *Leptospira rpoB* sequence entries in GenBank due to its use in many studies [30,31]. Previous research using serology to identify and name infecting *Leptospira* organisms (serovars) had major limitations. Although serology can reflect the epidemiology of circulating serovars, it does not identify species conclusively [29]. Furthermore, there is poor correlation between *Leptospira* serological (i.e., serovar) and genomic classification (i.e., species and strain), which complicates comparing current *Leptospira* genomic data with past and present serological data [29]. For instance, strains identified as belonging to serovar Bataviae have vast genetic heterogeneity, belonging to *L*. *borgpetersenii*, and *L*. *kirschneri*, *L*. *interrogans*, *L*. *noguchii*, *or L*. *santarosai*, according to genetic analyses [8]. *Leptospira* serological tests also have a history of inadequate sensitivity and specificity across a range of hosts, including instances where seronegative carriers had infectious leptospires in their urine and/or kidneys [32].

Accordingly, the purposes of this study are to determine whether bats in Grenada are actively infected with potentially pathogenic and possibly zoonotic species of *Leptospira* by PCR, determine genetic diversity of the leptospiral strains carried by bats through gene sequence analysis, and evaluate whether infection is associated with any gross or microscopic pathology. These data will provide insight into the diversity of bat-associated zoonotic leptospires in a tropical setting and establish the basis for determining the role of bats in transmitting *Leptospira* to humans.

## Materials and methods

### Ethics statement

All protocols for trapping, handling and euthanizing bats were approved by the Institutional Animal Care and Use Committee (IACUC-14008-R) at St. George’s University, School of Veterinary Medicine and with consent from the Grenada Ministry of Agriculture, Forestry, Wildlife and Fisheries, Grenada, West Indies. Bats were trapped using mist nets and hand nets. Both of these methods were approved as humane by the Animal Care and Use Committee of the American Society of Mammologists [33].

### Bat trapping

During 2015–2017, 173 clinically healthy bats representing both sexes and three abundant bat genera—several species of *Artibeus*, *Glossophaga longirostris* (GL), and *Molossus molossus* (MM)—were identified by morphology [34]. Due to the changing taxonomic status of bats in the *Artibeus jamaicensis* complex of bats [35], all potential *Artibeus jamaicensis*, *Artibeus planirostris*, and *Artibeus schwartzi* bats in this study are collectively identified as *Artibeus* spp. and abbreviated as (AS) (for *Artibeus* spp.), while *Artibeus literatus* (AL) bats in this study are treated as a separate taxon from AS bats.

Several mist nets (avinet.com) were used per site to ensure adequate monitoring and prompt removal of the bats. Mist nets were not left unattended at any time. Additionally, mist nets were not used in areas of high winds, as wind may contribute to stress and entanglement of bats. Captured bats were removed from the nets immediately, and all mist nets were removed immediately after the trapping period had ended.

### Processing of bats

All processing was conducted with appropriate personal protective equipment (latex gloves, surgical masks and eyewear). Rabies virus has not been detected in bats in Grenada using viral detection by RT-PCR or direct immunofluorescence, but neutralizing antibodies to rabies virus have been observed previously [36]. Thus, all personnel handling the bats had completed the rabies vaccination series and demonstrated protective titers. Live bats were transported to the necropsy laboratory at St. George’s University, School of Veterinary Medicine (SGU SVM), Grenada, West Indies, in individual opaque cloth bags to prevent post-capture cross-contamination. Bats were euthanized in the necropsy lab using isoflurane followed by thoracotomy and cardiac exsanguination while under anesthesia. Tissue samples were stored in RNAlater at -20°C and formalin.

### PCR and sequencing

In 2017, DNA was extracted from 30 mg of kidney after tissue disruption in a bead-beater (Mini Beadbeater Biospec Products, Bartlesville, OK, USA) using QIAamp DNA Mini Kit spin columns (QIAGEN, Hilden, Germany) according to the manufacturer’s directions. Generic zoonotic *Leptospira*-specific primers for a ~600 bp region of the *rpoB* gene (beta-subunit of RNA polymerase) were used [31]. After electrophoresis in a 2% agarose gel, bands of the expected size were extracted and sent for direct Sanger sequencing. Resulting sequences were analyzed and edited using Chromas 2.6.4 and compared to known *rpoB* gene sequences in NIH-NCBI GenBank using the Basic Local Alignment Search Tool (BLAST). In cases of overlapping sequence data, cloning and transformation were performed on amplicons from those samples. DNA was extracted from several colonies, and PCR was conducted using primers T7 (5'-TAATACGACTCACTATAGG-3’) and SP6 (5'-GATTTAGGTGACACTATAG-3') in the plasmid which flank the inserted amplicon. Briefly, PCR was performed using 40 cycles with 55°C annealing and one-minute extension. Amplicons from this PCR were then electrophoresed, extracted from gels, sent to Sanger sequencing, edited, and compared to GenBank entries as described above. Bats were considered PCR-positive for *Leptospira* infection after sequencing confirmation.

### Phylogenetic analysis

Phylogenetic trees were constructed with bat-derived *Leptospira* sequences and *rpoB* sequences from all *Leptospira* species retrieved from GenBank (S1 Table) using MEGA X. Specifically, maximum-likelihood phylogenetic analysis was conducted with the Kimura’s 2-parameter nucleotide substitution model, gamma-distributed substitution rates, and an allowance for invariant sites (K2+G+I) and with 1000 bootstrapped replicates. Nodes with bootstrap confidence below 70% were condensed in the phylogenetic tree presented. Pairwise sequence alignments were obtained using MEGA X software (S2 Table).

### Histopathology

Bat liver and kidney tissues were fixed by immersion in 10% neutral buffered formalin, embedded in paraffin, sectioned at 4 μm, stained with Hematoxylin & Eosin (HE) and Warthin-Starry (WS) silver stain (kidneys only) using standard histological techniques, and examined by light microscopy by a board certified veterinary pathologist. Staining with WS is an established method for the detection of *Leptospira* spp. within tissue sections [37,38].

### Statistical analysis

Relative risk was calculated to critically examine the association between bats that tested positive for *Leptospira* and the presence of histopathological lesions in the kidney. Sensitivity and specificity values were calculated to analyze the utility of WS staining technique relative to PCR testing.

Prevalence of *Leptospira* was compared between the two positive bat taxa and across years. Comparisons between species were done using chi-squared tests, both year by year except where counts were less than five for a given year, and overall. Comparisons across years were done using Spearman’s rank correlation to establish increasing or decreasing trend.

## Results

### Characteristics of *Leptospira* positive and negative bats

**Fig 1:**
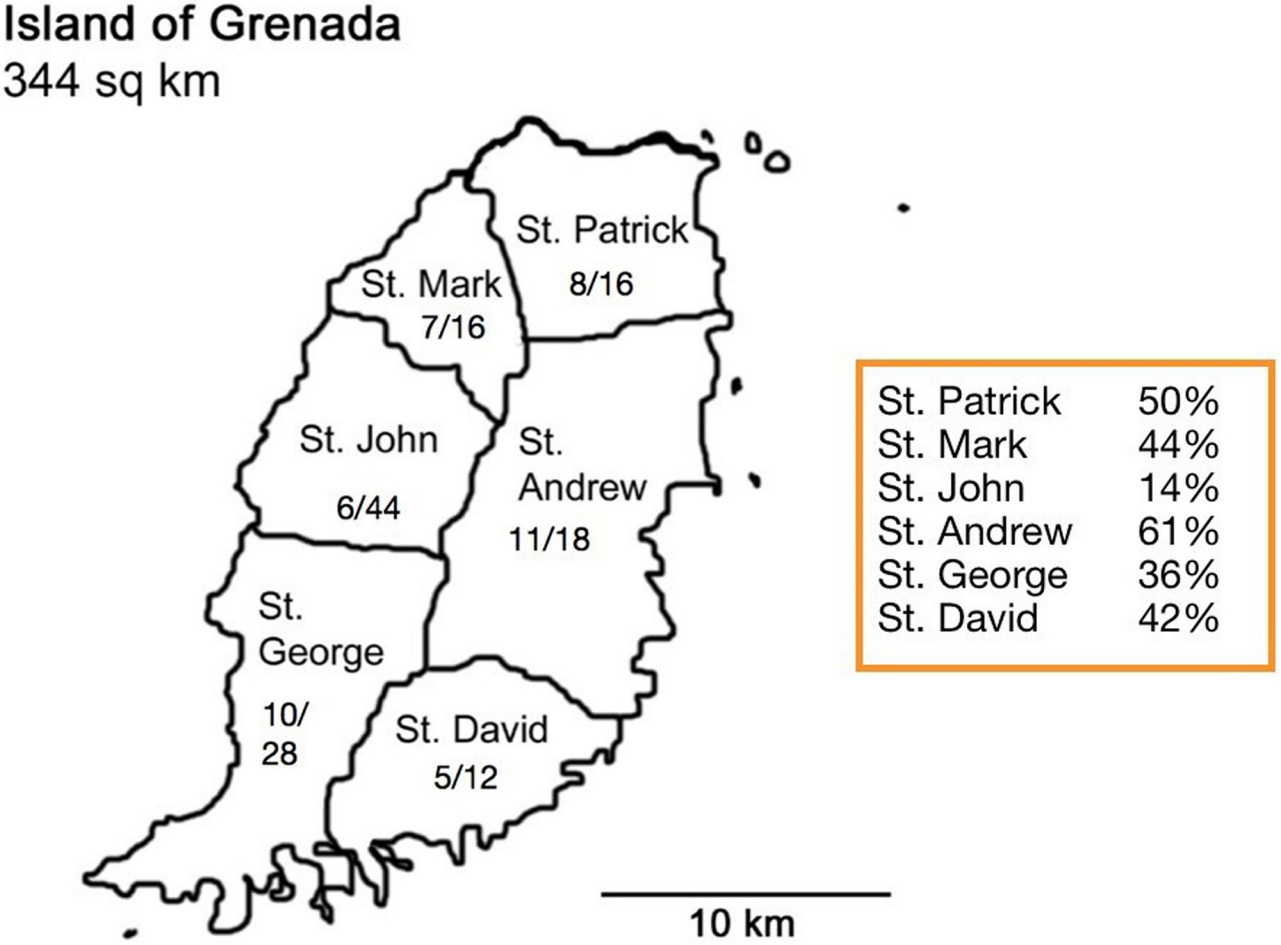
Map of Grenada. Distribution of bats PCR-positive for *Leptospira* by parish. Numbers on map represent: Bats positive/bats tested. Image adapted from: https://commons.wikimedia.org/wiki/File:Grenada_parishes_blank.png using Microsoft Powerpoint. The original image is licensed under the Creative Commons Attribution-Share Alike 3.0 Unported license.

The 173 bats evaluated in this study represent three genera: 51 *Glossophaga longirostris* (GL), 35 *Molossus molossus* (MM), 2 *Artibeus literatus* (AL), and 85 non-*Artibeus literatus* species of *Artibeus* (AS) (Table 1). Bats from all six parishes of Grenada were among the *Leptospira* PCR-positive (Fig 1). All *Leptospira* PCR-positive bats were either AS or GL (Table 1). Prevalence of *Leptospira* varied significantly among bat species. No MM or AL tested positive. Positive AS and GL bats were trapped in all three years of trapping (Table 1). Across all three years, significantly more GL bats were *Leptospira*-positive (33/51; 65%) than were AS bats (14/85; 16%) (p<0.0001). Furthermore, *Leptospira* infection rates in GL were significantly higher than those of AS in 2015, the only year in which both species were captured in abundance (GL: 31/43 [72%]; AS: 7/52 [13%]; p<0.0001.) Prevalence of *Leptospira* in GL bats decreased significantly (p = 0.01) from 72% in 2015 to 25% in 2016 and 2017; however, only 8 GL bats were caught in 2016–2017. Bats collected in both the dry and rainy seasons were found to be positive, but the prevalence of diseases did not differ between seasons (S1 Fig). Five of the 173 bats were pregnant, and three of them were PCR-positive for *Leptospira* (2 AS and 1 GL).

**Table 1:** Species of bats testing PCR-positive for *Leptospira* by year trapped.

Bat Species	2015 Positive/	2016*^ Positive/	2017* Positive/	Total Positive/	P for difference by year**
	Tested (%)	Tested (%)	Tested (%)	Tested (%)	
*Artibeus lituratus*	0/2 (0%)	-	-	0/2 (0%)	--
*Artibeus* spp.	7/52 (13%)	2/17 (12%)	5/16 (31%)	14/85 (16%)	0.20
*Glossophaga longirostris*	31/43 (72%)	1/4 (25%)	1/4 (25%)	33/51 (65%)	0.01
*Molossus molossus*	0/26 (0%)	-	0/9 (0%)	0/35 (0%)	--
Total:	38/123 (31%)	3/21 (14%)	6/29 (21%)	47/173 (27%)	0.11
P for difference by species, AS vs. GL***	<0.0001	0.50	0.81	<0.0001	

*Artibeus lituratus was not collected in 2016/2017.

^*Molossus molossus* was not collected in 2016.

**P for difference in *Leptospira* infection rates for each species, and then for total overall years, from Spearman’s rank correlation (P for increasing or decreasing trend over time).

***P for difference in *Leptospira* infection rates by species for each year, and then for total over all species, from chi-squared test (P that at least one species differs from at least one other).

--Chi-squared test was not used for comparing *Artibeus lituratus* or *Molossus molossus* differences by year because bats caught have expected values below 5.

### PCR-based identification and phylogenetic analysis of *Leptospira* spp.

Consensus sequences of over 450 bp of the *rpoB* gene were obtained from forward and reverse reactions from 31 of 47 *Leptospira*-positive samples. These Grenada bat derived *Leptospira* sequences were 87–91% identical to known *Leptospira rpoB* gene sequences in GenBank (Table 2). Isolate GBL-AS-x6 matches best with *Leptospira* sp. strain ADMAS 2667, a pathogen isolated from an infected dog in India for which the serovar or species was not determined. The best matches for the other 30 isolates are known pathogenic *Leptospira* species, including species known to cause disease in humans (*L*. *noguchii*, *L*. *santarosai*) [5].

**Table 2:** Best matches to bat derived *Leptospira rpoB* gene sequences found in GenBank.

Bat Species	Bat *Leptospira* ID number	Species and serovar of closest match in GenBank	Accession number of closest match	Percent identity	Accession number of *Leptospira* isolate
*Artibeus spp*.	GBL-AS-x3	*Leptospira kmetyi* strain LS 001/16	CP033614.1	497/548(91%)	MG981094
	GBL-AS-x4			486/548(89%)	MG981095
	GBL-AS-x9			482/548(88%)	MG981099
	GBL-AS-x30	*Leptospira mayottensis* strain MDI222	CP030144.1	485/548(89%)	MG981109
	GBL-AS-x7			493/557(89%)	MG981097
	GBL-AS-x8			432/488(89%)	MG981098
	GBL-AS-x2	*Leptospira noguchii* strain Cascata	EU349502.1	483/548(88%)	MG981093
	GBL-AS-x29	*Leptospira santarosai* strain U160	CP027843.1	476/548(87%)	MG981108
	GBL-AS-x6	*Leptospira* sp. ADMAS 2667	JN388649.1	498/548(91%)	MG981096
*Glossophaga longirostris*	GBL-GL-x1*	*Leptospira kmetyi* strain LS 001/16	CP033614.1	427/479(89%)	MG981092
	GBL-GL-x15			492/548(90%)	MG981101
	GBL-GL-x18			492/548(90%)	MG981103
	GBL-GL-x23			492/548(90%)	MG981106
	GBL-GL-x24			491/548(90%)	MG981107
	GBL-GL-x33			497/548(91%)	MG981111
	GBL-GL-x34			459/517(89%)	MG981112
	GBL-GL-x36			484/548(88%)	MG981113
	GBL-GL-x37			492/548(90%)	MG981114
	GBL-GL-x38			460/517(89%)	MG981115
	GBL-GL-x47			492/548(90%)	MG981117
	GBL-GL-x48			492/548(90%)	MG981118
	GBL-GL-x51			492/548(90%)	MG981119
	GBL-GL-x73*			381/426(89%)	MG981120
	GBL-GL-x75			485/548(89%)	MG981121
	GBL-GL-x76			492/548(90%)	MG981122
	GBL-GL-x14	*Leptospira kmetyi* strain LS 001/16 *Leptospira mayottensis* strain MDI272 *Leptospira mayottensis* 200901116	CP033614.1 CP030147.1 CP024871.1	482/548(88%)	MG981100
	GBL-GL-x16	*Leptospira mayottensis* strain MDI222	CP030144.1	485/548(89%)	MG981102
	GBL-GL-x20			485/548(89%)	MG981105
	GBL-GL-x45	*Leptospira noguchii* strain Cascata	EU349502.1	467/522(89%)	MG981116
	GBL-GL-x19			491/548(90%)	MG981104
	GBL-GL-x32			483/548(88%)	MG981110

GenBank accession numbers and percent identity between the bat derived *Leptospira rpoB* gene sequence and its best match in GenBank, as determined by BLAST, are provided. For all sequences, the percent identity was calculated using over 99% of the Grenada bat *Leptospira*-derived sequences.

Samples with asterisks (bats GBL-GL-x1 and GBL-GL-x73) were not used in subsequent phylogenetic analyses because of degenerate nucleotides in the sequence data.

**Fig 2:**
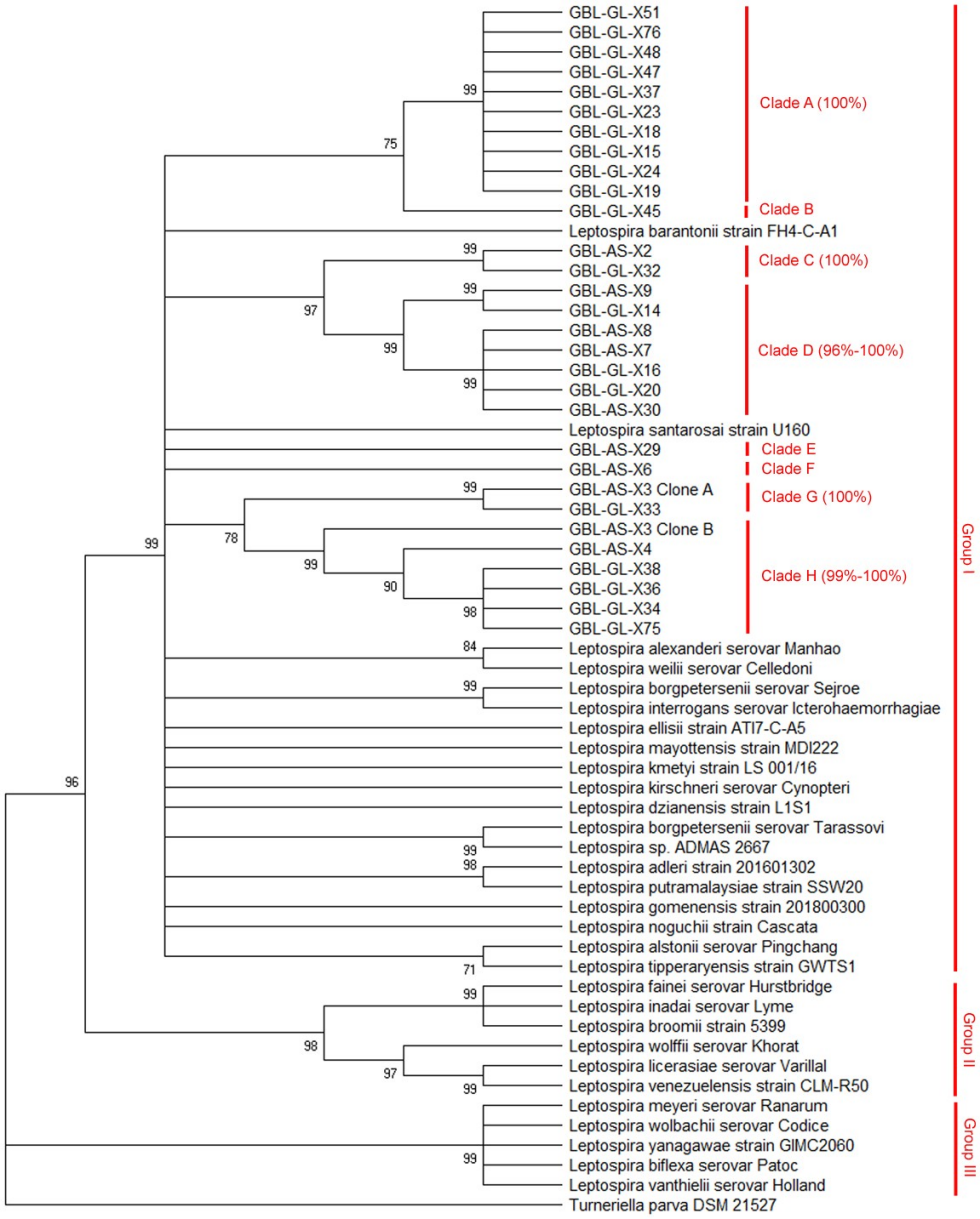
Phylogenetic tree representing the relationships among *Leptospira* identified in this study (70% bootstrap cutoff). Maximum-likelihood phylogenetic trees were derived from partial *rpoB* gene sequences in GenBank for known species and by sequencing of partial *rpoB* gene from Grenadian bat-derived *Leptospira* spp. sequences. Bootstrap values are listed at each node. Nodes with bootstrap confidence values below 70% support are condensed. Clusters representing potentially novel *Leptospira* taxa are designated Clades A-H in the tree. Within each group, the range for the percent identities of each pairwise comparison is shown next to the group name in parentheses. Labels: GBL—Grenada Bat *Leptospira*; AS—*Artibeus* spp. complex bat; GL—*Glossophaga longirostris*; x##—specimen number.

Pairwise comparisons of all the Grenada bat derived *Leptospira* sequences ranged from 79–100% (S2 Table). The bat-derived *Leptospira* identified herein likely comprise several *Leptospira* species; this is corroborated by comparing overlapping regions of the Grenada bat-derived *Leptospira rpoB* gene sequences with known *Leptospira* sequences in GenBank using MEGA X (Fig 2).

All Grenadian bat *Leptospira* isolates fall within the pathogenic branch and form eight discrete clusters that are distinct from all previously identified *Leptospira* serovars. We designated these clusters Clades A-H (Fig 2) based upon their similarity to each other: specifically, for each bat-derived isolate, it is (A) 96% or more identical to all other isolates within its clade and (B) 92% or less identical to any other isolates outside of its clade used in the phylogenetic analysis.

### Histopathological examination of bat tissue

In general, all of the examined bats appeared to be healthy based on postmortem examination, e.g., adequate body condition, mild to moderate parasite burdens, no lesions that suggest significant overt disease within the examined organ systems.

Liver and kidney sections from 124 bats in this study were examined for histopathological lesions that may be associated with leptospirosis. Mild or moderate chronic non-suppurative interstitial nephritis was observed in 29/124 (23%) of the examined bats. Inflammatory lesions accompanied by renal tubular degeneration and necrosis, indicative of mild tubulointerstitial nephritis, was observed in 6/124 (5%) of the examined bats. Relative risk (RR) was calculated to examine the association between PCR-positive bats and the presence of histopathological lesions in the kidney. The probability of a wild captured bat to have interstitial nephritis and be PCR-positive for *Leptospira* is 24% with a RR of 31%. Thus, PCR-positivity for *Leptospira* is not strongly associated with the presence of interstitial nephritis.

**Fig 3:**
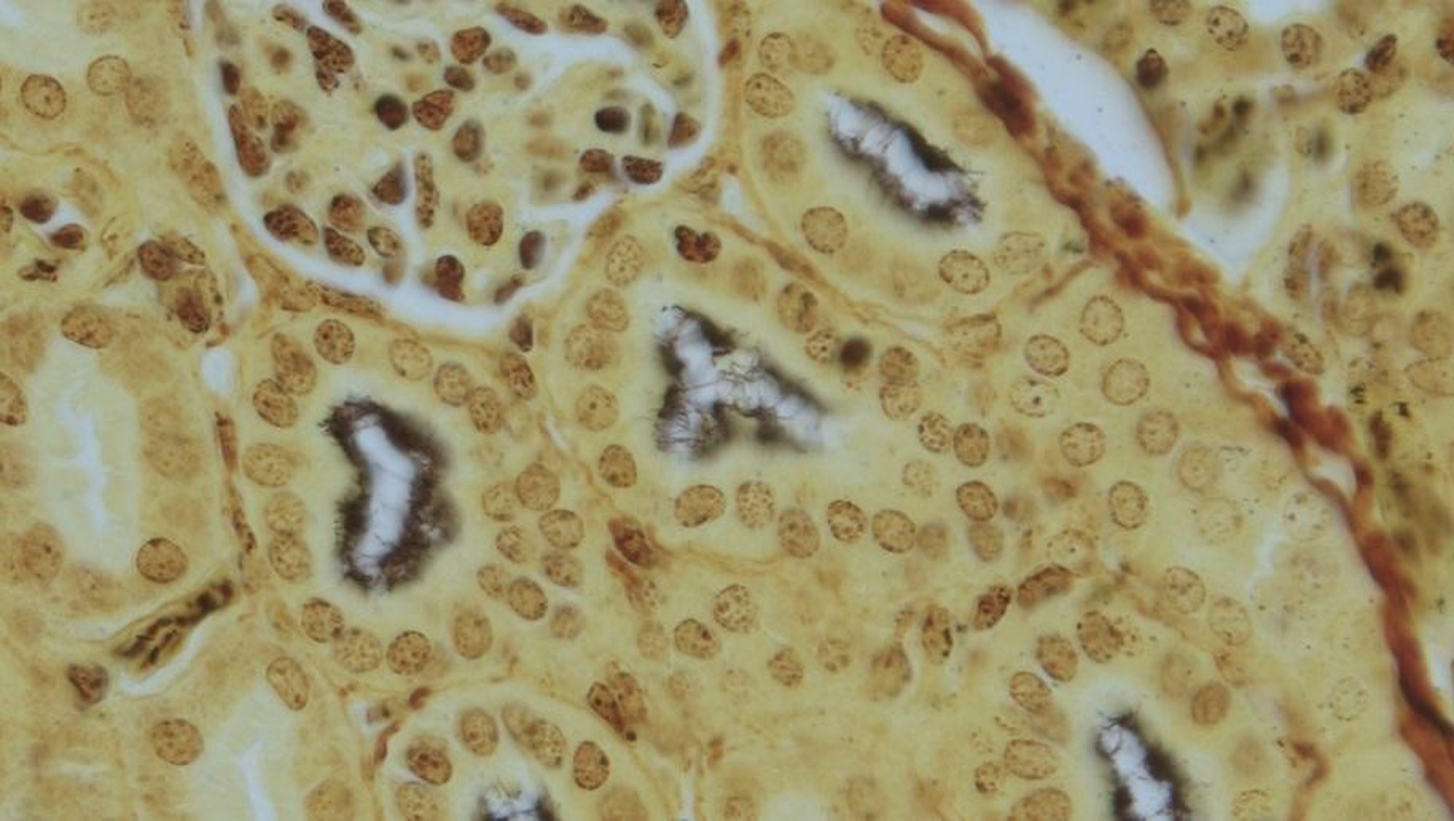
Bat kidney histology. The luminal surface of the renal tubular epithelial cells are multifocally colonized by numerous black leptospires. Warthin-Starry. 40X objective.

Warthin-Starry (WS) silver stain was applied to the kidney sections of 44 bats that tested PCR-positive for *Leptospira* with 31/44 (70%) testing WS-positive (Fig 3). Conversely, WS stain was applied to the kidneys of five bats that tested PCR-negative for *Leptospira* which resulted in 0/5 (0%) WS positives. Thus, the WS staining technique performed with 70% sensitivity and 100% specificity when compared with conventional PCR methods used in this study.

## Discussion

The overall prevalence of *Leptospira* PCR-positive bats in this study (27%) was considerably higher than the 12% seroprevalence previously reported in Grenada bats. Although four taxa of bats were sampled in our study, only two were *Leptospira* PCR-positive: *Artibeus* spp. (AS) and *Glossophaga longirostris* (GL). Our findings in these two bat species are in agreement with reports detecting of *Leptospira* antigen or antibody reported in various countries including Brazil [39], Peru [15,18], and Grenada and Trinidad and Tobago [11]. We found 65% of GL bats were *Leptospira* PCR-positive, which is approximately eight-fold higher than the seroprevalence rate previously reported for this species of bats from Grenada (8%) [11]. In our study, only two *Artibeus literatus* (AL) bats were evaluated, both trapped in the same year and both *Leptospira* PCR-negative. While the negative findings may reflect the small number of AL bats tested, another study that sampled 22 bats of this species also failed to detect any *Leptospira* positive animals [15]. However, *Leptospira* PCR-positive AL bats have been reported in at least one study from Mexico (2/8 positive; 25%) [19]. The other bat species that tested *Leptospira*-negative in our study was *Molossus molossus*. This species represented 26 of 173 bats trapped and included bats from two of the three trapping years. *Leptospira* PCR-positive *M*. *molossus* have previously been reported at low rates in Brazil (4/19 bats positive; 21%) and Trinidad (5/20; 25%) [11,39]. Accordingly, we may have missed finding *Leptospira*-positive MM bats simply due to the low number of captured MM and possibly low infection rates overall in this species. Also, of five pregnant bats collected, three tested *Leptospira* positive. Though this is a small sample size, this may reflect increased *Leptospira* exposure and/or susceptibility of pregnant bats, as has been reported elsewhere [40].

Importantly, *Leptospira* infection rates differed greatly between AS and GL bats (16% vs. 65%). Bat *Leptospira* infection rates based on molecular techniques or culture from other studies also show marked variation from 0% to over 80% depending on bat species and location [14–17,20,22,39–41]. Some speculate that the primary bat feeding habits (fruit, nectar, and insect) represented in most studies also explain some of the infection rate differences, but no statistically significant data have been published to confirm this. Grenada is an entirely semi-rural country, with no large densely populated urban centers. All the bats trapped in our study lived in close proximity to human homes, roosting in covered porches of lived-in houses, abandoned structures, and in nearby orchards, forests, and caves. Thus, location, time of year and age of bat, together with *Leptospira* detection methods could have impacted observed infection rates in this study.

The closest match in GenBank for each of the *Leptospira*-positive samples belonged to Group I (i.e., pathogenic *Leptospira*). Based on recommendations by La Scola et al. (2006), *Leptospira* sequences with *rpoB* identity lower than 92% represent different species, and 97–100% identity between partial *rpoB* gene sequences suggests that isolates are conspecific [31]. All of the bat derived *Leptospira* partial *rpoB* sequences in this study were less than 92% identical to known *Leptospira rpoB* gene sequences in GenBank (Table 2) (range of best match identity: 87–91%). Thus, *Leptospira* genotypes described herein are presumably different species from all *Leptospira* with *rpoB* gene sequences catalogued in GenBank. Furthermore, our analysis suggests that the Grenada bats tested are infected with as many as eight separate undescribed *Leptospira* taxa, in which each genotype within a clade is 97–100% identical to all other members of its clade and ≤92% identical to other *Leptospira* in this study and in GenBank (Fig 2, S1 Table). Pairwise sequence comparison of overlapping regions, presented in S1 Table as percent identity, between each pair of Grenada bat derived *Leptospira rpoB* gene sequences demonstrated a 79–100% identity. Furthermore, for our phylogenetic analysis, we included at least one partial *rpoB* sequence from each of the pathogenic/Group I *Leptospira* species [4,42] and none are conspecific to the bat *Leptospira* isolates described herein by percent identity or phylogenetic analysis. Thus, based on the La Scola et al. (2006) recommendations, Grenadian bats harbor several distinct genotypes of *Leptospira*. Other studies have similarly demonstrated that even within a limited geographical range, bats are often infected with a diverse range of *Leptospira* [15,19,20] including potentially novel strains [16,43]. However, while the DNA sequences from PCR and phylogenetic analysis merely suggest that bats in Grenada have genetically diverse genotypes, of which several are possibly novel strains, DNA sequences alone are not considered sufficient to classify our unique bat-derived *Leptospira* genotypes reported herein as novel strains or species. DNA-DNA hybridization and more thorough genomic sequencing are necessary before we can deem our bat-derived *Leptospira* as novel taxa [4,29].

Clade H, the only genotype detected all three years of the study, was only detected in one bat in two of the three years surveyed (Table 3). Clade A, the predominant genotype in GL in 2015, was found in five of six parishes that year, but was not detected in 2016 or 2017. Annual changes in the dominant *Leptospira* serovars or strains have been observed in other studies of *Leptospira*-positive animals in the Caribbean islands [44] and elsewhere [45,46] but the prevalence changes observed over time in our study may also reflect the relatively small number of positive bats analyzed.

**Table 3:** Grenada bat *Leptospira* clade by year and bat species.

Grenada Bat *Leptospira* Clade	2015	2016	2017
**Clade A**	10 GL		
**Clade B**	1 GL	1 GL	
**Clade C**	1 GL	1 AS	
**Clade D**	1 AS, 3 GL		3 AS
**Clade E**	1 AS		
**Clade F**			1 AS
**Clade G**	1 AS	1 GL	
**Clade H**	1 AS, 4 GL	1 AS	1 AS

AS–*Artibeus* spp. complex; GL–*Glossophaga longirostris*

In addition to bats, other animals in Grenada (cane toads, cattle, mongoose, and sheep) also have reacting antibodies to *L*. *noguchii* serovar Bataviae and *L*. *borgpetersenii* serovar Tarassovi [11,26,27], while Grenadian cane toads, cattle, chickens, mongooses, and pigs have reacting antibodies to *L*. *santarosai* serovar Shermani [11,26,27]. Several bats in this study produced *Leptospira* sequences that matched with *L*. *noguchii* and *L*. *santarosai*, albeit at low identities (below 90%). However, serology identifies serotypes and does not correlate with species in typing *Leptospira* [8,29], and hence we cannot compare our current genomic data with past serological data with any certainty. Thus, it is necessary to confirm *Leptospira* strains by PCR in these and other animals going forward in order to clarify *Leptospira* ecology and reservoir hosts in Grenada.

Interstitial nephritis and tubulointerstitial nephritis are renal lesions that are generally associated with leptospirosis. However, these findings, especially when mild, may also be nonspecific lesions that are commonly observed in many wildlife species and may have no direct correlation with leptospirosis. We conclude that bats are not likely to be adversely affected by *Leptospira* infection, based on the lack of association between PCR-positivity for *Leptospira* and the presence of renal inflammatory lesions (RR = 31%). These results may also reflect that renal lesions of *Leptospira*-infected bats are transient and may not be detectable throughout the duration of chronic infection, similar to what has been described in rats, where subclinical chronic infection is marked by mild inflammatory renal lesions and no overt disease [47].

This is the first study to demonstrate the colonization of renal tubules by leptospires in wild captured bats using light microscopy with WS staining technique. Colonization of the renal tubules is a prerequisite for transmission of leptospires in the urine, and our findings provide further evidence that bats may indeed be an important reservoir host for zoonotic leptospirosis with potential for spread to humans via urine transmission. Future studies in experimental bat models are needed to determine the efficiency of urine shedding and transmission of *Leptospira* from bats to other individuals which will provide a better understanding of the zoonotic potential and public health risks posed by bats.

This study is the first report of molecular detection of *Leptospira* in bats in Grenada. Importantly, this is also the first report of phylogenetic analysis of *Leptospira* detected in any species of animal or humans from Grenada and a starting point for future comparative studies to improve our understanding of the epidemiology of *Leptospira*. Results show that Grenada bats are infected with novel and diverse *Leptospira* genotypes phylogenetically related to known pathogenic, including zoonotic, taxa. Further, our results suggest that infected bats are asymptomatic with concomitant renal *Leptospira* colonization that can be shed in urine. Together, these findings reinforce bats’ roles as potential reservoirs of *Leptospira*.

## Supporting information

S1 Table GenBank accession numbers for the *rpoB* gene sequences of *Leptospira* spp. and *Turneriella* spp. bacteria used in phylogenetic analysis of Grenada Bat *Leptospira* relatedness. (XLSX)

S2 Table Pairwise alignments of Grenada bat *Leptospira*. Thirty-one *Leptospira* spp. isolates with at least 450 bp sequences were compared by pairwise sequence alignment using MEGA X. Values are presented as percent identity.
(XLSX)

S3 Table A Fisher’s exact test was performed to determine the significance of the number of *Leptospira* PCR positive bat tissues in the rainy season (July to December) compared to the dry season (January to June). There is not a significant difference between the number of positive *Artibeus* spp. and positive *Glossophaga longirostris* infected with *Leptospira* in the rainy season compared to the dry season (p = 0.1258). The analysis for *Molossus molossus* were excluded because of 0 values. Labels; AS–*Artibeus* spp. complex; GL–*Glossophaga longirostris*; MM–*Molossus molossus*.
(XLSX)

S1 Fig Number of *Leptospira* PCR positive bat tissues in the rainy season compared to the dry season. (TIFF)
