# Supplementary figures and images for: Phylogenetic relationships and diversity of bat-associated Leptospira and the histopathological evaluation of these infections in bats from Grenada, West Indies
Source: PLoS Negl Trop Dis. 2020 Jan 21;14(1):e0007940. doi: 10.1371/journal.pntd.0007940 (PMC6994174; doi:10.1371/journal.pntd.0007940)

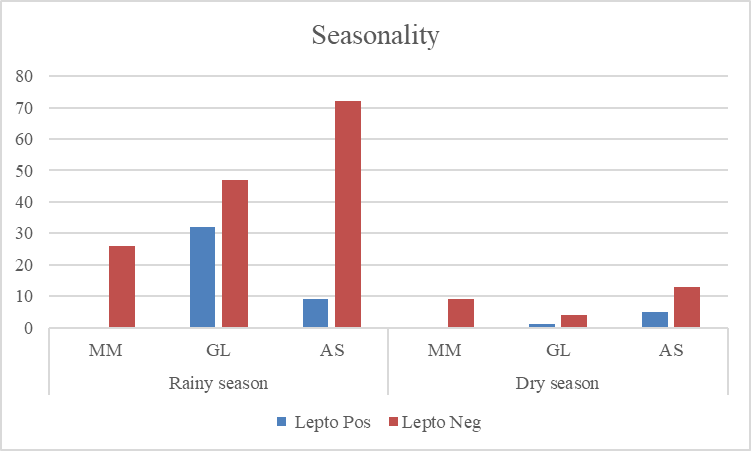

Supplement: S1 Fig — (TIFF) [file pntd.0007940.s004.tiff]
